# SpatialPEFT: a parameter-efficient fine-tuning framework for spatial transcriptomics foundation models

**DOI:** 10.1093/bioinformatics/btag503

**Published:** 2026-07-08

**Authors:** Xin Zou, Xiujuan Lei

**Affiliations:** School of Artificial Intelligence and Computer Science, Shaanxi Normal University, Xi'an, Shaanxi 710119, China; School of Physics and Electronic Information Engineering, Ningxia Normal University, Ningxia, Guyuan 756000, China; School of Artificial Intelligence and Computer Science, Shaanxi Normal University, Xi'an, Shaanxi 710119, China

## Abstract

**Summary:**

SpatialPEFT is a unified parameter-efficient fine-tuning framework that enables the robust adaptation of large spatial transcriptomics foundation models (up to 1.4 billion parameters) on a single 16 GB consumer-grade GPU. By integrating Low-Rank Adaptation (LoRA), gradient checkpointing, and a spatial-aware adapter, it reduces peak VRAM by over 87% while substantially improving downstream spatial annotation accuracy.

**Availability and implementation:**

SpatialPEFT is implemented in Python and released under the MIT license. The source code, documentation, and tutorials are freely available at https://github.com/applerplay/SpatialPEFT, with an archival snapshot deposited at Zenodo (DOI: 10.5281/zenodo.20725321).

## 1 Introduction

Spatial transcriptomics has reshaped the landscape of gene expres-sion profiling while maintaining native tissue structure, which facilitates direct investigation of cellular organization in situ. To analyze such data, single-cell foundation models (scFMs), such as Geneformer (Theodoris *et al.* 2023), scGPT (Cui *et al.* 2024), and UCE (Rosen *et al.* 2023), have demonstrated remarkable transfer learning capacity across diverse downstream tasks. However, fine-tuning these models—especially large variants with up to 1.4 billion parameters—typically requires institutional-grade GPUs with 32 GB or more of VRAM. This static and activation memory footprint creates a prohibitive computational barrier for most academic laboratories. Such a constraint is especially limiting for spatial transcriptomics, where adapting foundation models to tissue-specific contexts could substantially improve downstream analyses such as spatial domain identification and cell type annotation.

Furthermore, existing PEFT tools such as scPEFT (He *et al.* 2026) were designed exclusively for dissociated single-cell data and lack three capabilities essential for spatial transcriptomics: (i) native parsing of spatial platforms (Visium, Xenium, MERFISH, CosMx) with spatial coordinate extraction; (ii) a spatial-aware adapter that fuses (x, y) coordinate information directly with transcriptomic embeddings; and (iii) unified support for heterogeneous weight serialization formats across multiple foundation models, including fused QKV architectures incompatible with standard HuggingFace APIs. To address this gap, we present SpatialPEFT, the first PEFT framework explicitly designed to democratize the fine-tuning of spatial transcriptomics foundation models on consumer-grade hardware.

## 2 Implementation and features

**Figure 1:**
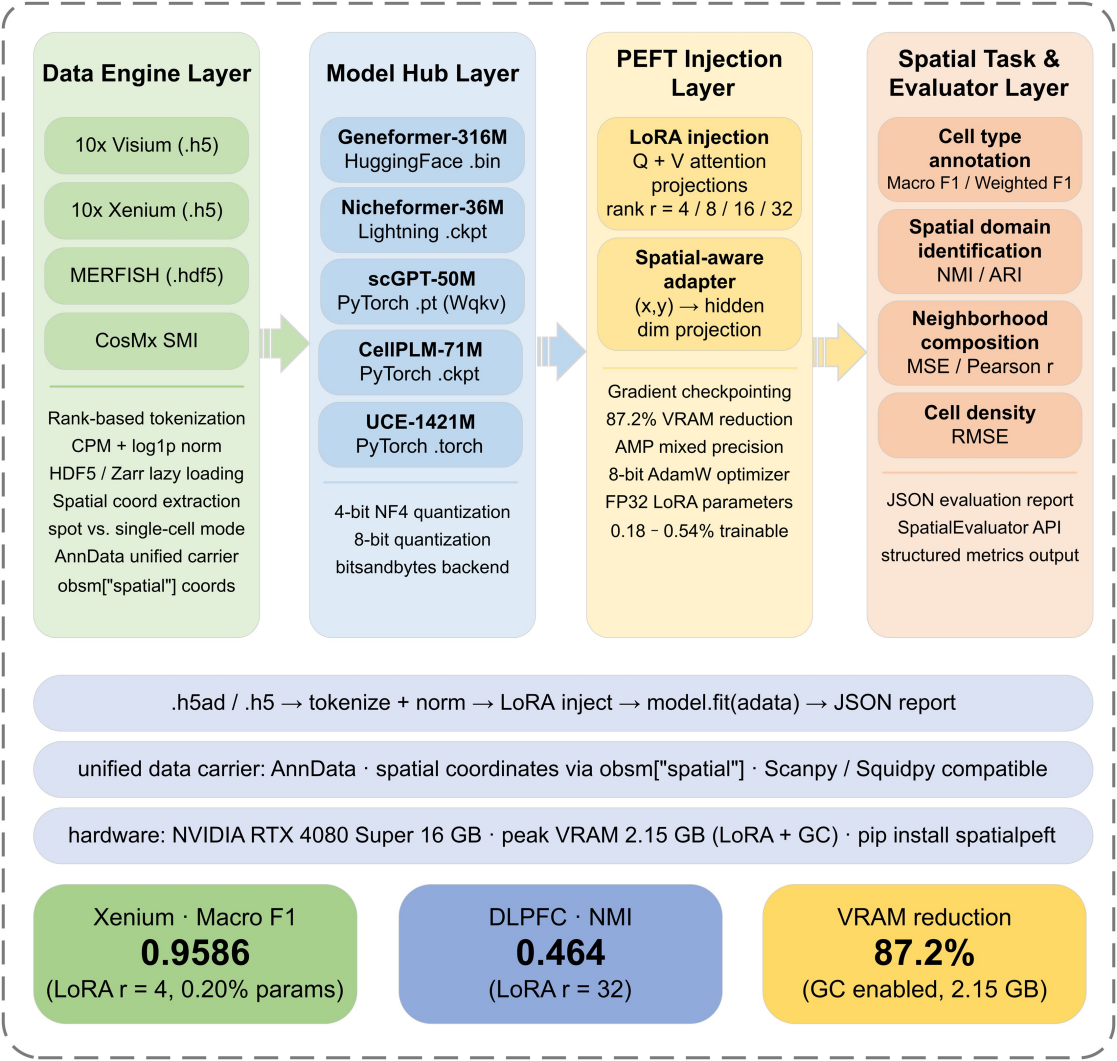
The modular architecture of SpatialPEFT. Data flows from the Data Engine Layer (preprocessing and tokenization) through the Model Hub Layer (quantized foundation models) to the PEFT Injection Layer (LoRA and spatial-aware adapter), culminating in the Spatial Task & Evaluator Layer for diverse downstream evaluations.

SpatialPEFT adopts a unified modular architecture built upon the AnnData ecosystem (Virshup *et al.* 2024), ensuring interoperability with standard spatial analysis pipelines (Palla *et al.* 2022) (Fig. 1).

### 2.1 Data engine layer

Natively parses data from spatial platforms including 10× Visium (Ståhl *et al.* 2016), Xenium (Janesick *et al.* 2023), MERFISH (Chen *et al.* 2015), and CosMx (He *et al.* 2022). It standardizes library-size normalization and log1p transformation, and converts each cell’s expression profile into a ranked token sequence by ordering genes according to normalized expression, so that the most informative genes appear first. For large-scale datasets, it implements batch-wise lazy loading via HDF5/Zarr backends.

### 2.2 Unified model hub layer

Harmonizes heterogeneous weight serialization formats (e.g. HuggingFace.bin, PyTorch.pt, Lightning.ckpt) to support five major scFMs: Geneformer, Nicheformer (Tejada-Lapuerta *et al.* 2025), scGPT, CellPLM (Wen *et al.* 2023), and UCE. It seamlessly integrates bitsandbytes for 4-bit and 8-bit quantized loading.

### 2.3 PEFT injection layer

Implements model-agnostic Low-Rank Adaptation (LoRA) (Hu *et al.* 2022, Mao *et al.* 2025), accommodating two distinct attention parameterizations. For models with fused QKV weight matrices (Nicheformer, scGPT, and UCE), SpatialPEFT manually slices the packed tensor to isolate the query and value sub-matrices before injecting low-rank updates, whereas for models exposing independent projection layers (CellPLM) the LoRA modules replace the query and value projections directly. Crucially, a novel spatial-aware adapter—a lightweight linear projection—maps the normalized (*x, y*) coordinate pair into the model’s hidden dimension, and its output is added element-wise to the transcriptomic embedding, allowing spatial context to modulate the representation without altering the frozen backbone.

## 3 Application and performance

### 3.1 Experimental setup

Unless otherwise noted, all experiments were conducted on a single NVIDIA RTX 4080 Super (16 GB) using Geneformer-316M as the backbone, with LoRA applied to the query and value projections, a learning rate of 5 × 10^−5^, mixed-precision (AMP) training, and gradient checkpointing enabled. The Xenium cell type annotation experiment utilized a sequence length of 256, batch size of 32, and 3 training epochs. DLPFC spatial domain identification used the same configuration on the standard 8-slice-train/4-slice-test split. Annotation quality was quantified by Macro and Weighted F1, and spatial domain agreement by Normalized Mutual Information (NMI) and Adjusted Rand Index (ARI), each computed against expert or marker-based reference labels.

### 3.2 Memory efficiency

**Figure 2:**
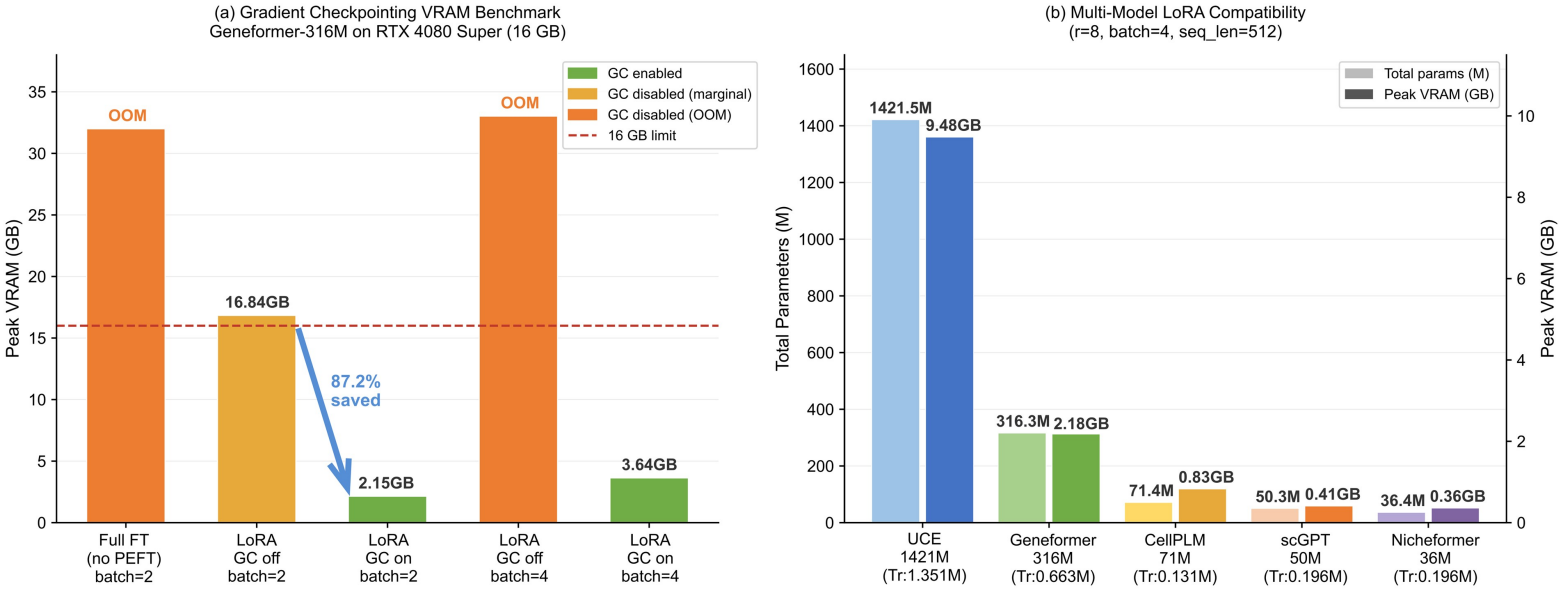
VRAM efficiency and multi-model compatibility on a 16 GB GPU. (a) Peak VRAM consumption of Geneformer-316M, illustrating the memory reduction achieved by gradient checkpointing (GC). (b) Peak VRAM and total parameter counts across five supported spatial foundation models during LoRA fine-tuning (*r *= 8, batch = 4).

We benchmarked VRAM requirements as follows. Full fine-tuning of Geneformer-316M at batch size 2 exceeds 32 GB (Out-of-Memory). This heavy memory footprint arises because the process requires storing optimizer states, gradients, and intermediate activations for all 316 million model parameters. By updating fewer than 1% of parameters through LoRA and trading activation storage for on-demand recomputation via gradient checkpointing, SpatialPEFT drastically reduces peak VRAM to 2.15 GB (an 87.2% reduction), scaling efficiently to batch size 4 using only 3.64 GB (Fig. 2a). Furthermore, LoRA compatibility was successfully validated across all five supported models. Peak VRAM remains well within the 16 GB budget for all evaluated models, including the 1.4B-parameter UCE model (9.48 GB) (Fig. 2b).

### 3.3 Biological validation

SpatialPEFT was evaluated on two complementary spatial datasets. On the 10× Xenium FFPE human breast cancer dataset (576 342 single cells, 11 classes), SpatialPEFT-LoRA (*r *= 4) achieved a Macro F1 score of 0.9586, improving over zero-shot inference (0.7104) by 24.8 percentage points using only 0.20% trainable parameters. On the spot-level DLPFC 12-slice dataset (47 329 Visium spots, 7 cortical layers) (Maynard *et al.* 2021), SpatialPEFT achieved an ARI of 0.382, a 177% relative improvement over zero-shot (ARI = 0.138). While this remains below purpose-built spatial GNN methods that explicitly construct neighborhood graphs for spatial domain identification (e.g. stHGC (Wang  *et al.* 2025) and graph contrastive clustering (Zhang *et al.* 2024); see also a recent review (Wang  *et al.* 2024)), this gap is expected, as Geneformer was pre-trained on dissociated single-cell data without spatial context; SpatialPEFT nonetheless demonstrates effective spatial awareness injection.

To illustrate the fine-tuned model’s spatial resolution, we visualized the eleven annotated cell populations across the Xenium tissue section (Fig. S1, available as supplementary data at *Bioinformatics* online). The full-section view reveals a heterogeneous tumor microenvironment in which cancer subtypes occupy the majority of the tumor parenchyma, while cancer-associated fibroblasts and immune cells are interspersed throughout; a zoomed-in view further highlights the fine-grained spatial interleaving of multiple cancer subtypes with cancer-associated fibroblasts. Resolving these morphologically similar, co-localizing populations is inherently difficult using zero-shot encoder representations, underscoring the necessity and effectiveness of task-specific fine-tuning via SpatialPEFT.

### 3.4 Rank sensitivity

A rank sensitivity analysis revealed a dataset-dependent saturation behavior: single-cell resolution data (Xenium) saturates at *r *= 4, whereas spot-level data (DLPFC) benefits from a higher rank of *r *= 16 due to overlapping cellular signals within capture spots. A 5-seed robustness analysis confirmed that increasing the rank from *r *= 16 to *r *= 32 yields no statistically significant improvement (NMI 0.457 ± 0.010 vs 0.463 ± 0.005, Welch’s *t*-test *P *= 0.26), indicating that *r *= 16 is sufficient for spot-level tasks (Supplementary Table S4, available as supplementary data at *Bioinformatics* online). This dataset-dependent behavior reflects the intrinsic task complexity at each resolution level. For single-cell Xenium data with well-separated cell lineages, the pre-trained Geneformer representations already span a subspace amenable to simple low-rank adaptation, so *r *= 4 suffices to redirect this subspace toward the target task. In contrast, each DLPFC Visium spot aggregates signals from 5 to 15 cells across morphologically similar cortical layers (particularly L2 and L3), creating a substantially more ambiguous feature space that benefits from a richer adaptation subspace—although the five-seed analysis confirms this benefit saturates by *r *= 16.

To further verify that these conclusions do not depend on the specific train/test split, we performed leave-one-slice-out cross-validation across the four held-out DLPFC slices, using each in turn as the test set while training on the remaining eleven (Table S5, available as supplementary data at *Bioinformatics* online). Performance was highly consistent across the four folds (NMI 0.5648 ± 0.0159, ARI 0.5097 ± 0.0196); because each fold trains on more slices than the main benchmark, the absolute values are higher and serve to quantify cross-partition variance rather than absolute accuracy. Together, the five-seed and leave-one-slice-out analyses establish that SpatialPEFT is robust to both random initialization and data partition choice.

Detailed benchmark configurations, multi-model gradient verifications, rank sensitivity analyses, seed- and partition-level robustness analyses, and high-resolution spatial visualizations are provided in Supplementary Sections S1–S10, Supplementary Figures S1–S2, and Supplementary Tables S1–S7, available as supplementary data at *Bioinformatics* online.

## 4 Discussion

SpatialPEFT effectively resolves the hardware bottleneck hindering the widespread deployment of spatial foundation models. By reducing VRAM requirements by over 87% without compromising downstream spatial annotation accuracy, it democratizes access to state-of-the-art AI models, enabling resource-constrained laboratories to conduct scalable spatial transcriptomics research on consumer-grade hardware. This accessibility is underpinned by a model-agnostic design that handles heterogeneous checkpoint formats across five architectures spanning 36M to 1.4B parameters, allowing future spatial transcriptomics foundation models to be integrated without bespoke engineering.

Two limitations merit note. First, the Xenium cell type labels were derived from unsupervised Leiden clustering followed by marker-gene annotation rather than orthogonal experimental validation, so the reported annotation accuracy should be interpreted relative to this reference. Second, while LoRA compatibility was verified across all five foundation models, full downstream benchmarking was concentrated on Geneformer; extending complete spatial task evaluation to the remaining models is a natural next step. Beyond these, adapting the framework to other data modalities—such as spatial proteomics—offers a promising direction for extending parameter-efficient fine-tuning across the broader landscape of spatial omics.

## Author contributions

Xin Zou (Conceptualization [Equal], Methodology [Equal], Software [Lead], Data Curation [Lead], Investigation [Lead], Formal Analysis [Lead], Visualization [Lead], Writing—original draft [Lead], Writing—review & editing [Supporting]), and Xiujuan Lei (Conceptualization [Equal], Methodology [Equal], Supervision [Lead], Funding Acquisition [Lead], Project Administration [Lead], Writing—original draft [Supporting], Writing—review & editing [Lead])

## Supplementary Material

btag503_Supplementary_Data

## Data Availability

The datasets analyzed in this study are publicly available. The 10x Xenium FFPE Human Breast Cancer dataset is available from 10x Genomics (https://www.10xgenomics.com/datasets). The DLPFC 12-slice Visium dataset (Maynard *et al.* 2021) is available via the spatialLIBD Bioconductor package. All source code is available at https://github.com/applerplay/SpatialPEFT and archived at Zenodo (DOI: 10.5281/zenodo.20725321).

## References

[btag503-B1] Chen KH , Boettiger AN, Moffitt JR et al Spatially resolved, highly multiplexed RNA profiling in single cells. Science 2015;348:aaa6090. 25858977 10.1126/science.aaa6090 PMC4662681

[btag503-B2] Cui H , Wang C, Maan H et al scGPT: toward building a foundation model for single-cell multi-omics using generative AI. Nat Methods 2024;21:1470–80. 38409223 10.1038/s41592-024-02201-0

[btag503-B3] He F , Fei R, Krull JE et al Harnessing the power of single‑cell large language models with parameter‑efficient fine‑tuning using scPEFT. *Nat Mach Intell* 2026;8:118–33. 10.1038/s42256-025-01170-z

[btag503-B4] He S , Bhatt R, Brown C et al High-plex imaging of RNA and proteins at subcellular resolution in fixed tissue by spatial molecular imaging. Nat Biotechnol 2022;40:1794–806. 36203011 10.1038/s41587-022-01483-z

[btag503-B5] Hu EJ , Shen Y, Wallis P et al LoRA: low-rank adaptation of large language models. ICLR 2022;1:3.

[btag503-B6] Janesick A , Shelansky R, Gottscho AD et al High resolution mapping of the tumor microenvironment using integrated single-cell, spatial and in situ analysis. Nat Commun 2023;14:8353. 10.1038/s41467-023-43458-x. 38114474 PMC10730913

[btag503-B7] Mao Y , Ge Y, Fan Y et al A survey on LoRA of large language models. Front Comput Sci 2025;19:197605.

[btag503-B8] Maynard KR , Collado-Torres L, Weber LM et al Transcriptome-scale spatial gene expression in the human dorsolateral prefrontal cortex. Nat Neurosci 2021;24:425–36. 33558695 10.1038/s41593-020-00787-0 PMC8095368

[btag503-B9] Palla G , Spitzer H, Klein M et al Squidpy: a scalable framework for spatial omics analysis. Nat Methods 2022;19:171–8. 35102346 10.1038/s41592-021-01358-2 PMC8828470

[btag503-B10] Rosen Y , Roohani Y, Agarwal A et al Universal cell embeddings: a foundation model for cell biology. *BioRxiv* 2023. 2023.11.28.568918.

[btag503-B11] Ståhl PL , Salmén F, Vickovic S et al Visualization and analysis of gene expression in tissue sections by spatial transcriptomics. Science 2016;353:78–82. 27365449 10.1126/science.aaf2403

[btag503-B12] Tejada-Lapuerta A , Schaar AC, Gutgesell R et al Nicheformer: a foundation model for single-cell and spatial omics. Nat Methods 2025;22:2525–38. 41168487 10.1038/s41592-025-02814-z PMC12695652

[btag503-B13] Theodoris CV , Xiao L, Chopra A et al Transfer learning enables predictions in network biology. Nature 2023;618:616–24. 37258680 10.1038/s41586-023-06139-9 PMC10949956

[btag503-B14] Virshup I , Rybakov S, Theis FJ et al Anndata: access and store annotated data matrices. Joss 2024;9:4371.

[btag503-B15] Wang R , Dai Q, Duan X et al stHGC: a self-supervised graph representation learning for spatial domain recognition with hybrid graph and spatial regularization. Brief Bioinform 2025;26:bbae666. 10.1093/bib/bbae666 PMC11663487 39710435

[btag503-B16] Wang Z , Geng A, Duan H et al A comprehensive review of approaches for spatial domain recognition of spatial transcriptomes. Brief Funct Genomics 2024;23:702–12. 39426802 10.1093/bfgp/elae040

[btag503-B17] Wen H , Tang W, Dai X et al CellPLM: pre-training of cell language model beyond single cell. *BioRxiv* 2023. 2023.10.03.560734.

[btag503-B18] Zhang Y , Yu Z, Wong KC et al Unraveling spatial domain characterization in spatially resolved transcriptomics with robust graph contrastive clustering. Bioinformatics 2024;40:btae451. 39012523 10.1093/bioinformatics/btae451 PMC11272174

