# Supplementary material for: SpatialPEFT: a parameter-efficient fine-tuning framework for spatial transcriptomics foundation models
Source: Bioinformatics. 2026 Jul 8;42(7):btag503. doi: 10.1093/bioinformatics/btag503 (PMC13395097; doi:10.1093/bioinformatics/btag503)
Supplement: btag503_Supplementary_Data [file btag503_supplementary_data.docx]

**Supplementary Material**

*SpatialPEFT: A Parameter-Efficient Fine-Tuning Framework for Spatial Transcriptomics Foundation Models*

**S1. Software Environment and Reproducibility**

## S1.1 Hardware Specification

| **Component** | **Specification** |
| --- | --- |
| CPU | Intel Core i9-14900KF |
| System RAM | 64 GB DDR5 |
| GPU | NVIDIA GeForce RTX 4080 Super |
| GPU VRAM | 16,376 MiB (≈16 GB GDDR6X); PyTorch reports 17.2 GB |
| Operating System | Windows 11 + WSL2 (Ubuntu 22.04) |
| CUDA Driver | 560.94 (Windows) / 560.35.02 (WSL2) |
| CUDA Runtime | 12.6 |
| Storage | NVMe SSD (required for lazy-loading large datasets) |

## S1.2 Software Versions

| **Package** | **Version** | **Purpose** |
| --- | --- | --- |
| Python | 3.10.x | Runtime |
| PyTorch | 2.5.1 (cu121) | Deep learning backend |
| transformers | 4.40.0 | HuggingFace model loading |
| peft | 0.10.0 | LoRA / PEFT injection |
| bitsandbytes | 0.49.2 | 4-bit / 8-bit quantization |
| scanpy | 1.9.x | Single-cell data processing |
| anndata | 0.9.x | AnnData format |
| scikit-learn | 1.3.x | Label encoding, metrics |
| matplotlib | 3.7.x | Figure generation |

## S1.3 Installation Commands

conda create -n spatialpeft python=3.10 -y

conda activate spatialpeft

# Install PyTorch with CUDA 12.1 support

pip install torch==2.5.1 torchvision torchaudio --index-url https://download.pytorch.org/whl/cu121

pip install transformers==4.40.0 peft==0.10.0 accelerate

pip install bitsandbytes==0.49.2

pip install scanpy anndata scikit-learn matplotlib

# Set HuggingFace mirror (China mainland)

echo 'export HF_ENDPOINT=https://hf-mirror.com' >> ~/.bashrc

**S2. Dataset Details**

## S2.1 Dataset D1: Xenium FFPE Human Breast Cancer

| **Property** | **Value** |
| --- | --- |
| Full name | 10x Xenium FFPE Human Breast Cancer IDC with Custom Add-on Panel |
| Source | 10x Genomics (https://www.10xgenomics.com/datasets) |
| Technology | 10x Xenium (imaging-based, subcellular resolution) |
| Total cells (before QC) | 576,963 |
| Cells after QC (≥5 transcripts) | 576,342 |
| Targeted genes | 380 (Custom Add-on Panel) |
| Tissue type | FFPE human breast cancer, invasive ductal carcinoma (IDC) |
| Cell type annotation | Leiden clustering (resolution = 0.5) + marker gene annotation |
| Number of cell types | 11 |
| Train / Test split | 80/20 stratified (461,073 train, 115,269 test) |
| Spatial coordinates | x_centroid, y_centroid (normalized to [0,1]) |

Cell type labels were assigned based on the following marker genes present in the Xenium panel (Note: MKI67 was not included in the Custom Add-on Panel; MDM2 was used as a surrogate proliferation marker.):

| **Cell type** | **Marker genes used** |
| --- | --- |
| Cancer cell (luminal-like) | FAM107B, NARS, MUC1, ANKRD30A, CLIC6 |
| Cancer-associated fibroblast | LUM, POSTN, AEBP1, CTHRC1, THBS2 |
| Cancer cell (GATA3+) | GATA3, MUC1, MDM2, ANKRD30A |
| T cell | PTPRC, CD3E, CD52, TRAC |
| Myeloid cell | FCER1G, LYZ, AIF1, CD4 |
| Cancer cell (secretory) | MUC1, CLIC6, SERPINA3 |
| Cancer cell (hormone+) | GATA3, SERPINA3, TPD52 |
| Endothelial cell | SPARCL1, AQP1, CAV1, RGS5 |
| Cancer cell (basal-like) | FAM107B, CLIC6, MUC1 |
| Myoepithelial cell | DST, MYLK, KRT14, SFRP1 |
| Cancer cell (proliferating) | MDM2, GATA3, MUC1 |

## S2.2 Dataset D2: DLPFC 12-Slice Human Brain

| **Property** | **Value** |
| --- | --- |
| Full name | Human Dorsolateral Prefrontal Cortex (DLPFC) 12-slice Visium |
| Source | Maynard et al. (2021), Nature Neuroscience; available via spatialLIBD |
| Technology | 10x Visium (sequencing-based, ~55 μm spot diameter, 5–15 cells/spot) |
| Slices | 12 (151507–151510, 151669–151672, 151673–151676) |
| Total labeled spots | 47,329 |
| Genes (after QC) | ~33,000 |
| Cell type annotation | Expert neuropathologist annotation (Maynard et al., 2021) |
| Labels | 7 (Layer1, Layer2, Layer3, Layer4, Layer5, Layer6, WM) |
| Note | Slices 151669–151672 lack Layer1 and Layer2 labels (known technical limitation) |
| Train split | 8 slices (151507–151510, 151669–151672): 33,086 spots |
| Test split | 4 slices (151673–151676): 14,243 spots |

**S3. Detailed Memory Efficiency Benchmarks**

Supplementary Table S1 benchmarks the peak VRAM of gradient checkpointing on the Geneformer-316M model. Without gradient checkpointing, LoRA fine-tuning with a batch size of 2 occupies 16.84 GB of memory, which slightly surpasses the 16 GB GPU memory ceiling and brings a high risk of out-of-memory (OOM) failures. Activating gradient checkpointing cuts the peak VRAM usage down to 2.15 GB, an 87.2% memory reduction; even when scaling the batch size up to 4, peak memory consumption only reaches 3.64 GB, corresponding to an 89.0% drop. By contrast, full fine-tuning without PEFT is computationally infeasible on our hardware: it would require an estimated 32+ GB of VRAM at batch size 2, far exceeding available GPU resources.

**Supplementary Table S1.** VRAM benchmarks for Geneformer-316M fine-tuning on RTX 4080 Super (16 GB).

| **Configuration** | **Batch size** | **Peak VRAM (GB)** | **Fits in 16 GB?** |
| --- | --- | --- | --- |
| Full fine-tune (no PEFT), GC off | 2 | ~32 GB (est.) | No — OOM |
| LoRA only, GC off | 2 | 16.84 | Marginal |
| **LoRA + GC on** | **2** | **2.15** | **Yes — 87.2% saved** |
| LoRA only, GC off | 4 | 33.03 | No — OOM |
| LoRA + GC on | 4 | 3.64 | Yes — 89.0% saved |

GC = gradient checkpointing. Full fine-tune estimate based on theoretical memory accounting. LoRA rank *r* = 8, targets: query and value projections.

**S4. Detailed Spatial Annotation Benchmarks**

## S4.1 Xenium Breast Cancer (Cell Type Annotation)

Supplementary Table S2 reports cell-type annotation performance on the Xenium test dataset, which comprises 115,269 cells across 11 distinct cell classes. Zero-shot prediction via the spatial linear probe yields a Macro F1 of 0.7104 and a Weighted F1 of 0.7303. Our SpatialPEFT-LoRA with rank *r* = 4 delivers the best performance with a Macro F1 of 0.9586, representing a 24.8 percentage point improvement over the zero-shot baseline. This variant only introduces 633,227 trainable parameters, accounting for merely 0.20% of the model’s total parameters, with a peak VRAM footprint of 2.17 GB. Raising the LoRA rank to *r* = 8 or *r* = 16 fails to further boost Macro F1 scores, demonstrating that predictive performance plateaus at *r* = 4 for this cell annotation task. Under the Xenium benchmark setup (sequence length = 256, batch size = 32), the zero-shot baseline consumes 1.20 GB VRAM, while LoRA *r* = 4 and LoRA *r* = 16 consume 2.17 GB and 2.82 GB respectively. All configurations stay well within the 16 GB GPU memory capacity with ample spare memory remaining.

**Supplementary Table S2.** Xenium FFPE breast cancer spatial cell type annotation (115,269 test cells, 11 classes).

| **Method** | **Macro F1** | **Weighted F1** | **Peak VRAM (GB)** | **Train time (min)** | **Trainable params** |
| --- | --- | --- | --- | --- | --- |
| Zero-shot + Spatial Probe | 0.7104 | 0.7303 | 1.20 | 78.5 | 301,451 |
| **SpatialPEFT-LoRA *r* = 4** | **0.9586** | **0.9574** | **2.17** | **273.9** | **633,227** |
| SpatialPEFT-LoRA *r* = 8 | 0.9528 | 0.9525 | 2.18 | 274.5 | 965,003 |
| SpatialPEFT-LoRA *r* = 16 | 0.9584 | 0.9573 | 2.82 | 272.5 | 1,628,555 |
| SpatialPEFT-LoRA *r* = 32 | 0.9576 | 0.9569 | 2.21 | 275.6 | 2,955,659 |

All experiments: RTX 4080 Super (16 GB), batch size 32, 3 epochs, seq_len 256. Best result highlighted. Dataset: 10x Xenium FFPE Human Breast Cancer IDC (576,342 cells, 380 genes). Cell type labels by Leiden clustering with marker gene annotation.

The spatial distribution of the eleven annotated cell populations is shown in Supplementary Figure S1, with panel (a) providing the full-section view and panel (b) a zoomed-in view of the tumor microenvironment; the biological interpretation is described in the main text (Biological Validation).

*
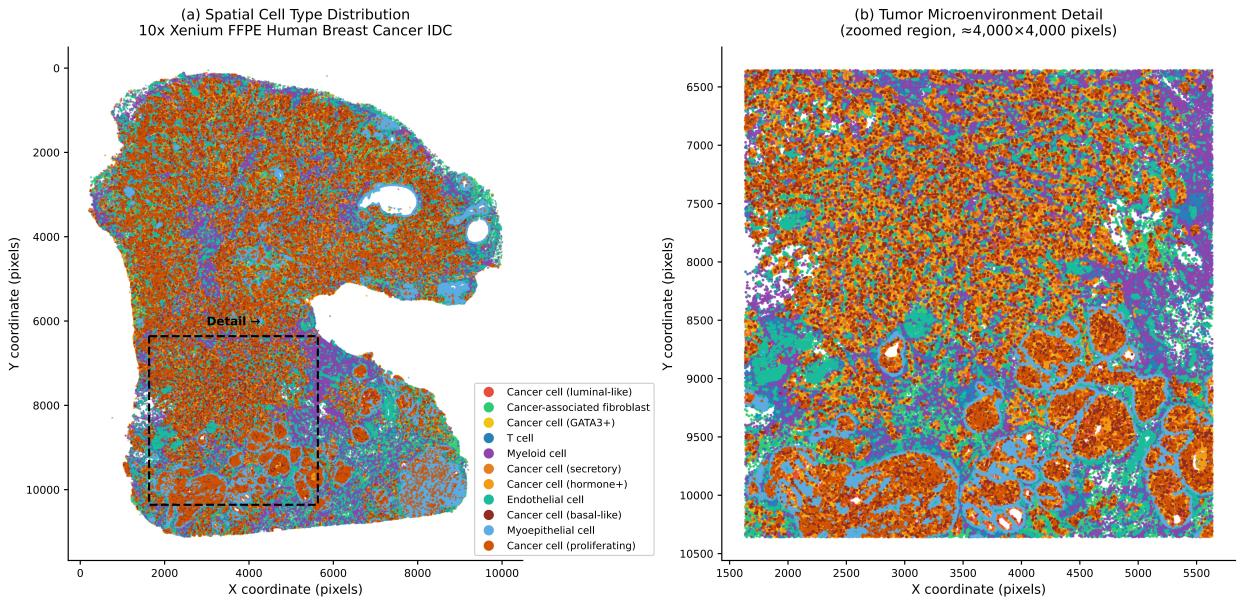
*

**Supplementary Figure S1.** Spatial cell type distribution in the Xenium FFPE human breast cancer dataset. (a) Full-section visualization of the 11 annotated cell populations across the tissue. (b) Zoomed-in detail of the tumor microenvironment showing the spatial interleaving of cancer cell subtypes and cancer-associated fibroblasts.

## S4.2 DLPFC Human Brain (Spatial Domain Identification)

Supplementary Table S3 summarizes spatial domain identification on the DLPFC test set (14,243 spots, 7 cortical layers, slices 151673–151676). Zero-shot inference achieves NMI = 0.300 and ARI = 0.1378, reflecting the difficulty of separating morphologically similar cortical layers from Visium spot-level mixed signals. SpatialPEFT-LoRA generally improves performance with increasing rank, with NMI improving from *r* = 4 (0.4369) to *r* = 16 and then plateauing (see Table S4 for the *r* = 16 vs *r* = 32 robustness analysis).

**Supplementary Table S3.** DLPFC 12-slice cortical layer identification (14,243 test spots, 7 layers).

| **Method** | **Macro F1** | **NMI** | **ARI** | **Peak VRAM (GB)** | **Train time (min)** | **Trainable params** |
| --- | --- | --- | --- | --- | --- | --- |
| Zero-shot + Spatial Probe | 0.2937 | 0.3000 | 0.1378 | 1.20 | 5.7 | 300,423 |
| SpatialPEFT-LoRA *r* = 4 | 0.4913 | 0.4369 | 0.3264 | 2.17 | 19.8 | 632,199 |
| SpatialPEFT-LoRA *r* = 8 | 0.5183 | 0.4540 | 0.3551 | 2.81 | 19.8 | 963,975 |
| SpatialPEFT-LoRA *r* = 16 | 0.5267 | 0.4594 | 0.3477 | 3.46 | 19.8 | 1,627,527 |
| **SpatialPEFT-LoRA *r* = 32** | **0.5423** | **0.4640** | **0.3824** | **4.12** | **19.7** | **2,954,631** |

Training: 8 slices (151507–151672), 33,086 spots. Test: 4 slices (151673–151676), 14,243 spots. Gold-standard labels from Maynard et al. (2021) expert neuropathologist annotation. Best result highlighted.Values in this table are from a single representative run (seed = 42). A 5-seed robustness analysis for *r* = 16 and *r* = 32 is provided in Supplementary Table S4, confirming that the difference between these two ranks is within seed variance (Welch's *t*-test *p* = 0.26 for NMI).

To assess the statistical reliability of the rank sensitivity results, we further evaluated the two highest ranks (*r* = 16 and *r* = 32) across five independent random seeds (0–4) under the fixed standard split, holding all other settings constant so that the only source of variation is the random seed. As shown in Table S4, the two ranks yield closely overlapping performance distributions (NMI 0.4569 ± 0.0101 for *r* = 16 versus 0.4632 ± 0.0049 for *r* = 32; ARI 0.3592 ± 0.0206 versus 0.3720 ± 0.0177). A Welch two-sample t-test reveals no statistically significant difference between the two ranks (NMI *p* = 0.26, ARI *p* = 0.32), indicating that the apparent single-run improvement from *r* = 16 to *r* = 32 falls within seed-level variance. We therefore conclude that *r* = 16 is sufficient for spot-level spatial domain identification, and that increasing the rank to *r* = 32 provides no statistically meaningful benefit while incurring additional parameter cost.

**Supplementary Table S4.** DLPFC LoRA rank robustness across 5 random seeds (fixed standard split, train 8 / test 4 slices).

| **Configuration** | **Seed** | **NMI** | **ARI** | **Macro F1** |
| --- | --- | --- | --- | --- |
| LoRA *r* = 16 | seed 0 | 0.4623 | 0.3885 | 0.5481 |
|  | seed 1 | 0.4655 | 0.3511 | 0.5116 |
|  | seed 2 | 0.4642 | 0.3726 | 0.4937 |
|  | seed 3 | 0.4499 | 0.3417 | 0.5305 |
|  | seed 4 | 0.4426 | 0.3421 | 0.5258 |
| **LoRA *r* = 16 (mean ± std)** | ***n* = 5** | **0.4569 ± 0.0101** | **0.3592 ± 0.0206** | **0.5219 ± 0.0205** |
| LoRA *r* = 32 | seed 0 | 0.4597 | 0.3751 | 0.5436 |
|  | seed 1 | 0.4606 | 0.3415 | 0.5063 |
|  | seed 2 | 0.4599 | 0.3750 | 0.5147 |
|  | seed 3 | 0.4711 | 0.3856 | 0.5532 |
|  | seed 4 | 0.4648 | 0.3827 | 0.5556 |
| **LoRA *r* = 32 (mean ± std)** | ***n* = 5** | **0.4632 ± 0.0049** | **0.3720 ± 0.0177** | **0.5347 ± 0.0227** |

All runs use Geneformer-316M, LoRA on query and value projections, seq_len 256, batch size 32, 3 epochs, lr 5×10⁻⁵, gradient checkpointing and AMP enabled, on an RTX 4080 Super (16 GB). The only variable across runs is the random seed. A Welch two-sample t-test between *r* = 16 and *r* = 32 yields *p* = 0.26 (NMI) and *p* = 0.32 (ARI), indicating the difference between *r* = 16 and *r* = 32 is not statistically significant; *r* = 16 is therefore sufficient for spot-level spatial domain identification.

To assess whether the reported spatial domain identification performance depends on the specific train/test partition, we performed leave-one-slice-out cross-validation on the four held-out DLPFC slices. In each fold, one of the four slices (151673, 151674, 151675, 151676) served as the standalone test set, and the remaining 11 slices were adopted for training. All other settings were identical to the main benchmark (Geneformer-316M, LoRA on query and value projections, *r* = 16, seq_len 256, batch size 32, 3 epochs, lr 5×10⁻⁵, gradient checkpointing and AMP enabled, seed = 42). The only variable differing across folds was the selected test slice.

**Supplementary Table S5.** DLPFC leave-one-slice-out cross-validation (*r* = 16, seed = 42).

| **Test slice** | **Train spots** | **Test spots** | **NMI** | **ARI** | **Macro F1** |
| --- | --- | --- | --- | --- | --- |
| 151673 | 43718 | 3611 | 0.5841 | 0.5387 | 0.6774 |
| 151674 | 43694 | 3635 | 0.5707 | 0.5003 | 0.6527 |
| 151675 | 43763 | 3566 | 0.5480 | 0.4956 | 0.6634 |
| 151676 | 43898 | 3431 | 0.5565 | 0.5043 | 0.6540 |
| **Mean ± SD** | **—** | **—** | **0.5648 ± 0.0159** | **0.5097 ± 0.0196** | **0.6619 ± 0.0111** |

Each fold uses one held-out slice as the test set and the remaining 11 slices for training. All settings match the main benchmark except the partition. Absolute values are higher than the 8-train/4-test main result (Table S3) due to the larger training set and are reported here to quantify cross-partition variance, not absolute accuracy.

**Important note on comparability.** Since each fold trains on 11 slices instead of the 8 slices used for the main benchmark (Table S3), the resulting absolute performance values are higher. These metrics are reported to characterize cross-partition variance rather than absolute accuracy, and the joint interpretation of this analysis and seed-level experiments is elaborated in the main text.

**S5. LoRA Rank Sensitivity Analysis**

Supplementary Figure S2 presents the rank sensitivity analysis across *r* = 4, 8, 16, 32 for both datasets. The results reveal a striking dataset-dependent saturation pattern. On Xenium (single-cell resolution), Macro F1 rises sharply from zero-shot (0.7104) to *r* = 4 (0.9586) and then plateaus with a maximum variation of 0.0058 across *r* = 4–32. Increasing rank from *r* = 4 to *r* = 32 multiplies the trainable parameter count by 4.7× (633K to 2,956K) yet yields no measurable accuracy gain. In contrast, on DLPFC (spot-level resolution), NMI improves from *r* = 4 (0.4369) to *r* = 16 and then plateaus; a 5-seed robustness analysis (Table S4) shows the difference between *r* = 16 and *r* = 32 is within seed variance (Welch's *t*-test *p* = 0.26).

The mechanistic interpretation of this dataset-dependent saturation—relating single-cell versus spot-level resolution to the required adaptation rank—is discussed in the main text (Application and Performance).

*
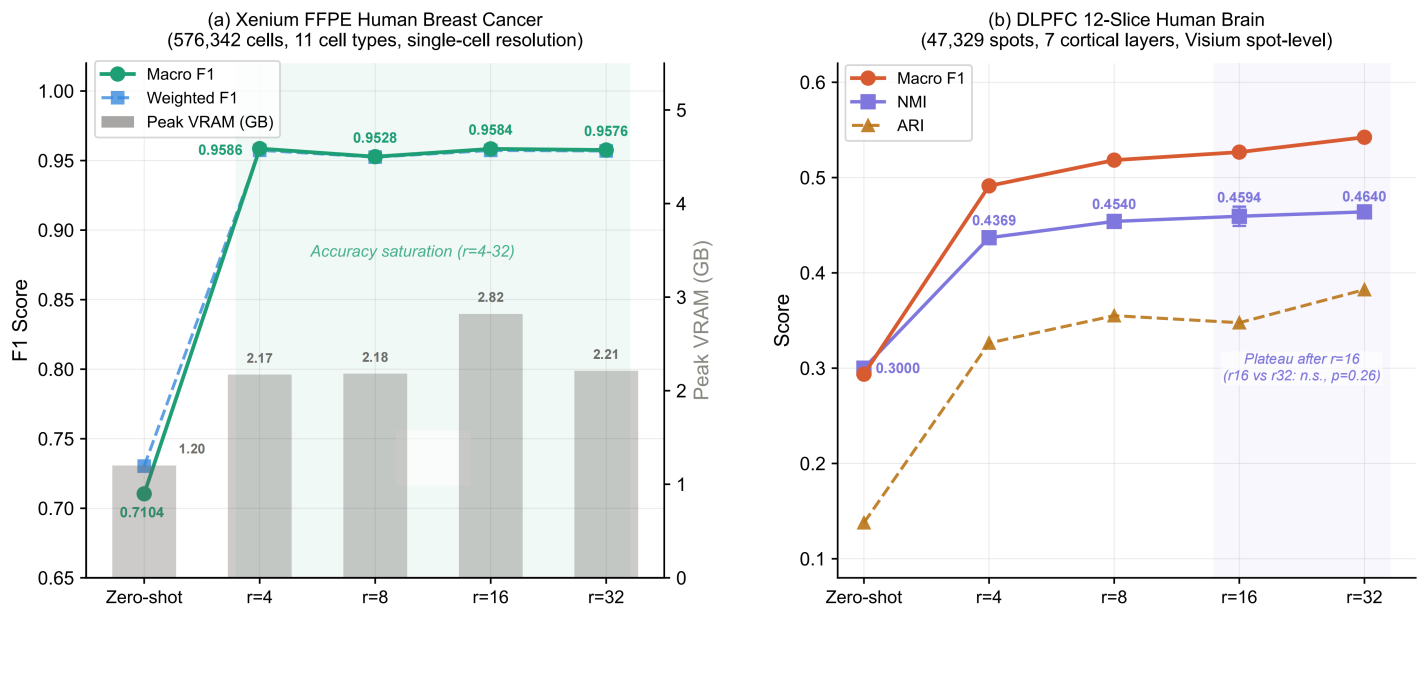
*

**Supplementary Figure S2.** Rank sensitivity curves for both benchmark datasets. (a) Xenium results, where Macro F1 rises sharply and then plateaus. (b) DLPFC results, where NMI improves from *r* = 4 to *r* = 16 and then plateaus. Key finding: Xenium shows accuracy saturation at *r* = 4 (single-cell, well-separated classes); DLPFC plateaus after *r* = 16, with no statistically significant difference between *r* = 16 and *r* = 32 (Welch's *t*-test *p* = 0.26).

**S6. Multi-Model LoRA Compatibility**

To validate SpatialPEFT’s model-agnostic design, we extended LoRA compatibility verification to five foundation models spanning four distinct weight formats and parameter scales. Geneformer (316.3M, HuggingFace .bin) serves as the primary experimental model. Nicheformer (36.4M, PyTorch Lightning .ckpt) and scGPT (50.3M, PyTorch .pt) use fused QKV weight matrices incompatible with the HuggingFace AutoModel API; SpatialPEFT’s manual QKV split strategy successfully injects LoRA on both. CellPLM (71.4M, PyTorch .ckpt) uses independent Q, K, V projection layers, enabling direct LoRA replacement. UCE (1,421.5M, PyTorch .torch) is the largest model verified, with the same fused in_proj_weight architecture as Nicheformer; LoRA injection yields 1,351,680 trainable parameters (0.205%) and 9.48 GB peak VRAM, remaining comfortably within the 16 GB hardware budget. All five models pass forward and backward pass validation with gradient verification (Supplementary Table S6).

**Supplementary Table S6.** Multi-model LoRA compatibility verification (*r* = 8, forward + backward pass).

| **Model** | **Params** | **Weight format** | **LoRA strategy** | **Trainable params** | **Trainable %** | **Peak VRAM** | **Status** |
| --- | --- | --- | --- | --- | --- | --- | --- |
| Geneformer-316M | 316.3M | HuggingFace .bin | HF PEFT standard | 663,552 | 0.21% | 2.18 GB | PASSED |
| Nicheformer-36M | 36.4M | Lightning .ckpt | Manual QKV split | 196,608 | 0.54% | 0.36 GB | PASSED |
| scGPT-50M | 50.3M | PyTorch .pt (Wqkv) | Manual QKV split | 196,608 | 0.39% | 0.41 GB | PASSED |
| CellPLM-71M | 71.4M | PyTorch .ckpt (model_state_dict) | Direct Q/V replacement | 131,072 | 0.184% | 0.83 GB | PASSED |
| UCE-1421M | 1,421.5M | PyTorch .torch (flat state dict) | Manual QKV split | 1,351,680 | 0.205% | 9.48 GB | PASSED |

All experiments: RTX 4080 Super (16 GB VRAM), seq_len = 512, batch = 4. Gradient check: all trainable parameters received non-NaN gradients after backward pass.

**S7. Technical Details for Non-Standard Model Checkpoints**

Geneformer adopts the standard Hugging Face PEFT API and does not require customized layer injection. Accordingly, the subsequent descriptions exclusively address the four non-standard checkpoints (Nicheformer, scGPT, CellPLM, and UCE).

## S7.1 Nicheformer LoRA Compatibility Technical Details

Nicheformer stores its pre-trained weights in PyTorch Lightning .ckpt format (a ZIP archive), with fused QKV attention weights in in_proj_weight per layer. SpatialPEFT's manual LoRA injection proceeds as follows:

(1) Load checkpoint: torch.load('nicheformer.ckpt', map_location='cpu')

(2) Extract state_dict keys matching encoder.*, embeddings.*, positional_embedding.*

(3) Reconstruct Nicheformer using PyTorch nn.TransformerEncoder

(4) In NicheformerLoRAAttention.forward(), split in_proj_weight into $W_{Q},W_{K},W_{V}$ and compute: $W_{Q\_eff}=W_{Q}+(\alpha/r)\times B_{Q}@A_{Q}$

(5) Freeze all non-LoRA parameters; cast LoRA matrices to FP32 for GradScaler compatibility.

## S7.2 scGPT LoRA Compatibility Technical Details

scGPT stores pre-trained weights in PyTorch .pt format, with fused QKV attention weights in Wqkv per layer. The weight mapping and LoRA injection strategy mirrors the Nicheformer approach by mapping Wqkv to in_proj_weight and splitting it manually before applying LoRA.The full step sequence follows S7.1, substituting the corresponding weight key (Wqkv for scGPT).

## S7.3 CellPLM LoRA Compatibility Technical Details

CellPLM stores pre-trained weights in PyTorch .ckpt format. Unlike Nicheformer and scGPT, CellPLM implements independent query_projection, key_projection, and value_projection Linear layers per encoder layer, enabling direct LoRA injection without QKV splitting by replacing the linear modules with LoRA wrappers.

## S7.4 UCE LoRA Compatibility Technical Details

UCE (Universal Cell Embeddings) stores pre-trained weights in PyTorch .torch format as a flat state dictionary. UCE uses a standard PyTorch TransformerEncoder with fused QKV weights in in_proj_weight per layer, identical in structure to Nicheformer. SpatialPEFT’s manual QKV split strategy therefore applies without modification.The full step sequence follows S7.1, substituting the corresponding weight key (in_proj_weight for UCE).

**S8. Supplementary Experiment: scRNA-seq Cell Type Annotation**

To demonstrate cross-data-type generalizability, we additionally benchmarked SpatialPEFT on a dissociated single-cell RNA-seq dataset (Human Breast Cancer, 45,647 cells). While this dataset lacks spatial coordinates, the experiment validates that the core LoRA injection mechanism functions equivalently on non-spatial transcriptomic data.

**Supplementary Table S7.** scRNA-seq cell type annotation benchmark (9,130 test cells, 13 classes).

| **Method** | **Macro F1** | **Weighted F1** | **Peak VRAM (GB)** | **Train time (min)** | **Trainable params** |
| --- | --- | --- | --- | --- | --- |
| Zero-shot + Linear Probe | 0.8424 | 0.8949 | 0.64 | 28.2 | 298,509 |
| SpatialPEFT-LoRA *r* = 8 | 0.9554 | 0.9630 | 2.70 | 96.8 | 962,061 |
| SpatialPEFT-LoRA *r* = 16 | 0.9520 | 0.9585 | 2.71 | 96.3 | 1,625,613 |

seq_len = 512, batch = 16, 5 epochs, learning rate 5×10⁻⁵ for LoRA. No spatial adapter used (dataset lacks coordinates). Results consistent with Xenium benchmark, confirming cross-data-type generalizability.

**S9. Known Issues and Solutions**

| **Issue** | **Cause** | **Solution** |
| --- | --- | --- |
| huggingface.co unreachable | Network blocked in China mainland | Set HF_ENDPOINT=https://hf-mirror.com |
| bitsandbytes CUDA Setup failed | Version incompatibility on WSL2 | Pin version: pip install bitsandbytes==0.49.2 |
| CUDA error: device-side assert | Token IDs exceed vocab_size | Ensure IDs < config.vocab_size (20275 for Geneformer) |
| OOM at batch = 2 without GC | Activation memory for long sequences (e.g. seq = 2048) | Enable model.gradient_checkpointing_enable() |
| LoRA loss = nan | FP16 gradients incompatible with GradScaler | Cast LoRA params to FP32 before optimizer step |
| ValueError: Attempting to unscale FP16 gradients | LoRA params stored in FP16 | for p in model.parameters(): if p.requires_grad: p.data = p.data.float() |
| nvcc not found | CUDA toolkit not installed | Harmless — PyTorch bundles its own CUDA runtime |
| TransformerEncoder batch_first AttributeError | Custom attn module missing batch_first | Add self.batch_first = True to custom attention class |

**S10. Code and Data File Inventory**

| **File** | **Description** |
| --- | --- |
| experiments/experiment_xenium.py | Xenium benchmark: Zero-shot + LoRA *r* = 4/8/16/32 (full 576K cells) |
| experiments/experiment_xenium_rank.py | Xenium rank sensitivity experiment |
| experiments/experiment_dlpfc.py | DLPFC benchmark: Zero-shot + LoRA *r* = 4/8/16/32 (12 slices) |
| experiments/test_nicheformer_lora.py | Nicheformer LoRA compatibility validation |
| experiments/test_scgpt_lora.py | scGPT LoRA compatibility validation |
| experiments/make_figures.py | Figure generation script (Figures 1–4) |
| experiments/test_geneformer.py | Geneformer model loading + forward pass validation |
| experiments/test_grad_checkpoint.py | Gradient checkpointing VRAM benchmark |
| experiments/experiment_scrna.py | scRNA-seq generalizability benchmark: Zero-shot (Supplementary S8) |
| experiments/experiment_scrna_lora.py | scRNA-seq generalizability benchmark: LoRA *r* = 8, *r* = 16 (Supplementary S8) |
| results/xenium_rank_results.json | Xenium rank sensitivity results |
| results/dlpfc_results.json | DLPFC benchmark results |
| results/nicheformer_compat.json | Nicheformer compatibility results |
| results/scgpt_compat.json | scGPT compatibility results |
| experiments/test_cellplm_uce_lora.py | CellPLM and UCE LoRA compatibility validation (Supplementary S6, S7.3-S7.4) |
| results/cellplm_uce_compat.json | CellPLM and UCE compatibility results |
